# The role of intrinsic motivation in sustaining citizen science participation among diverse participants in a corporate volunteer program

**DOI:** 10.1371/journal.pone.0331221

**Published:** 2025-09-05

**Authors:** Bradley C. Allf, Lincoln R. Larson

**Affiliations:** 1 Department of Applied Ecology, North Carolina State University, Raleigh, North Carolina, United States of America; 2 Department of Parks, Recreation & Tourism Management, North Carolina State University, Raleigh, North Carolina, United States of America; University of Tartu, ESTONIA

## Abstract

Citizen science enables volunteers from the public to contribute to scientific research. While citizen science may be an avenue for “democratizing” science and facilitating learning among volunteers, projects tend to attract homogeneous volunteers already highly engaged in science. The emergence of facilitator organizations such as schools, churches and corporations, that connect existing volunteer-oriented groups with citizen science, offers a potentially viable avenue through which to attract more diverse volunteers, with more to gain from their experience. However, attracting and retaining these volunteers requires a detailed understanding of their motivations, and how different types of motivations might facilitate sustained, impactful experiences. The objective of this study was to evaluate the motivations, engagement, and diversity of citizen science volunteers recruited through a corporate volunteer program. We assessed these outcomes using digital participation metadata and a survey of 388 employee volunteers. Over the course of three years (2019–2022), this program enabled over 13,000 volunteers to contribute approximately 31,000 hours to 49 citizen science projects, though approximately half of volunteers participated only once. Survey results indicated that most volunteers (85%) were new to citizen science, and were more representative of the general US population in terms of their race/ethnicity, gender and educational attainment than typical citizen scientists. Volunteers’ motivations were primarily self-determined (i.e., intrinsic). Less self-determined (i.e., extrinsic) motivations, like a sense of obligation or group pressure, were linked to lower levels of participation in the program. In addition, socio-demographic factors (race/ethnicity and age) were associated with differing levels of participation. In conclusion, volunteers from facilitator organizations can make significant contributions to citizen science and reshape citizen science into a more diverse and inclusive pursuit. However, sustained engagement by these volunteers relies in part on volunteers participating for intrinsic reasons. To facilitate self-determined motivation, projects should meet volunteers’ needs for competence, relatedness and autonomy.

## Introduction

Citizen science, one type of participatory science, is the practice of engaging volunteers in scientific research, typically through collecting or analyzing data to support scientific research run by institutions [1,2]. Citizen science projects exist across a wide spectrum of fields, from health-related projects like “Outbreaks Near Me” that track the spread of disease to biodiversity projects like “iNaturalist” that map and share biodiversity observations. Citizen science has grown in popularity over the past few decades, engaging more than a million volunteers every year in biodiversity monitoring alone [3,4]. Many of these volunteers participate in multiple different projects across disciplines [5]. Coinciding with this growth in popularity are new resources to support volunteers such as the website SciStarter.org, which serves as a project database for volunteers to find new projects as well as a hub for volunteer groups to organize their citizen science effort on curated web pages [6].

Citizen science holds significant scientific value for collecting and/or analyzing large datasets at broad spatiotemporal scales—datasets that would otherwise be too costly or otherwise inaccessible to scientists working on their own [7]. Citizen science also has value because it engages the public in scientific research, a characteristic which some have suggested could “democratize” science [8,9] as well as lead to volunteer learning, changes in volunteers’ behavior, and other outcomes [10,11]. For instance, participants in a project tracking indoor biodiversity reported gains in their interest in science after contributing to the project [12], and participants in an avian citizen science project increased their science literacy and sense of place as it relates to the environment [13]. Reviews of learning via citizen science suggest that gains in knowledge are the most commonly reported participant outcomes of citizen science, while changes in attitudes or behaviors are less commonly reported and more poorly understood [14].

### Conventional citizen scientists

One reason that citizen science projects rarely impact volunteers’ attitudes and behaviors is that volunteers often enter into projects already highly engaged with science and/or the environment and so are unlikely to show gains in these metrics; this phenomenon, often referred to as self-selection bias, can be compounded by a “ceiling effect” in measurement that makes differences in key outcome variables difficult to detect [15–18]. For instance, Brossard et al. [19] found that citizen scientists in the avian citizen science project “The Birdhouse Network,” were much more likely to express eco-centric, pro-conservation attitudes than people who weren’t citizen scientists; as a result, they concluded that “it may be difficult, and even unnecessary, to change project participants’ attitudes toward the environment” (p. 1113).

In addition to being more engaged in science and the environment than most people, the sociodemographic characteristics of citizen science volunteers also differ from those of people who are not citizen scientists. Specifically, with a few exceptions [20], when compared to the general public citizen scientists are more likely to be well-educated, relatively affluent, able-bodied, employed in science-related fields, and White [5,21–23]. To take just one particularly striking example, more than 99% of participants in a field-based avian citizen science project operating in nine countries in southern Africa identified as White [24].

The lack of diversity in citizen science has many negative consequences, including patchy data [21,25], the inequitable distribution of participation benefits [23,26], and a homogeneity of perspectives within the field of science [27].

### Facilitator organizations

One means of reaching new sources of citizen science volunteers is through “third party” or “facilitator” organizations already embedded in communities, such as schools, churches, corporate social responsibility programs, and community groups [10,23,26,28–32]. Because these organizations focus on volunteering or member engagement outside the specific context of science or the environment, their volunteers may have different characteristics than typical, self-selecting citizen science volunteers who independently seek out experiences with science and nature [28].

There are a growing number of facilitator organizations that engage volunteers in citizen science. For instance, libraries in the United States, Europe and the Middle East are now organizing successful opportunities for patrons to participate in citizen science [33,34]. Schools, another type of facilitator organization, are also engaging students in citizen science. For example, a camera trapping project has partnered with classrooms in India, Kenya, Mexico and the US to collect data on mammals [35], and an ecology project engaged primary and secondary school children in Austria in monitoring bees, hedgehogs, and other animals [36]. An environmental health citizen science project in the US state of Arizona engaged socio-demographically diverse volunteers in rainwater monitoring in part through partnerships with community health workers called promotoras [37]. An avian citizen science project called “Celebrate Urban Birds” successfully engaged diverse volunteers living in urban centers through community partnerships with faith-based groups, community centers, youth groups and rehabilitation programs [38].

One type of facilitator organization that has been little-studied in the context of citizen science participation (though see [28]) is corporate volunteer programs. The goal of these programs, which are one type of corporate social responsibility, is to encourage employees of a company to contribute time to their communities, non-profits or other charitable groups through planned volunteer activities [39]. These programs support important service activities, can improve employee morale, and may improve a company’s reputation [40]. Corporate volunteer programs sometimes involve citizen science. For instance, companies such as HSBC, Starbucks, Alcoa and Syngenta have partnered with the environmental charity Earthwatch to provide opportunities for cohorts of employees to work as field assistants for environmental scientists [41]. Partnerships such as these can have a positive impact on participants’ commitment to and efficacy for supporting sustainability initiatives at work and home [42,43]. Importantly, Earthwatch expeditions are uniquely immersive experiences within the context of citizen science, often costing hundreds of dollars and requiring international travel; most citizen science projects have far fewer barriers to entry.

### Motivations to participate

Some authors have pointed out that volunteers from facilitator organizations may have different motivations for participating in citizen science than typical volunteers who are often intrinsically motivated to participate [44,45]. For instance, students sometimes participate in citizen science because it is required by a school [46–49]. Other motivations potentially unique to facilitator organizations might include a member of a corporate volunteer program who is enticed to volunteer because prizes like paid time off are awarded to participating employees, a member of a church volunteer group who may feel group pressure to participate in a program, or Girl Scouts who join a citizen science program because their troop leader signs them up to participate [31]. Volunteers who seek out citizen science experiences independently of a facilitator organization are unlikely to share these group-oriented motives.

**Fig 1:**
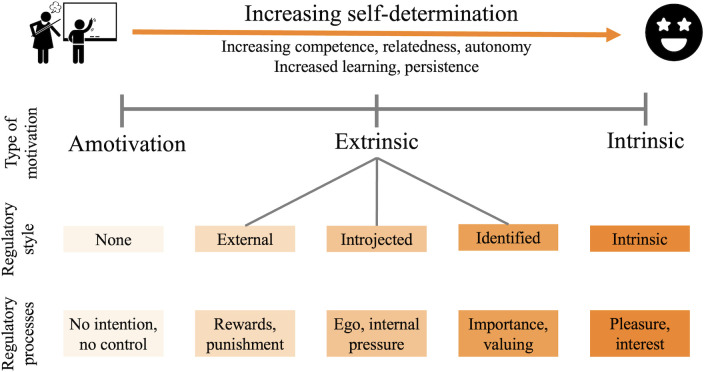
Motivations arranged along a spectrum of increasing self-determination. More self-determined motivations (i.e., those regulated by factors internal to the self) are facilitated by meeting individuals’ needs for competence, relatedness and autonomy and are linked to longer-lasting and more impactful behaviors. Figure adapted from Ryan & Deci [50] and does not include integrated regulation, which is sometimes situated between identified and intrinsic regulation.

Self-Determination Theory (SDT) is an empirical model of human motivation that is well-suited to addressing questions of motivations in citizen science volunteers from facilitator organizations [45]. SDT models motivation on a continuum of increasing “self-determination,” that is, from behaviors that one is compelled to do by external forces to behaviors whose motivations are experienced as emanating entirely from the self [50]. Specifically, SDT identifies different types of motivation along this continuum of increasing self-determination. The least self-determined motivation is amotivation—doing an activity despite any internal motivation to do so. Amotivation is followed, along the self-determination continuum, by extrinsic motivation. SDT identifies different types of extrinsic motivation embodying increasing levels of self-determination, from external regulation (doing things to get a reward or avoid punishment) to introjected regulation (doing things out of a sense of guilt or ego) to identified regulation (doing things because they are identified as important). The most self-determined motivation is intrinsic motivation—doing things for fully internalized reasons, such as because the behavior is simply enjoyable ([50]; Fig 1).

A robust body of literature, including in the contexts of education, health behavior change, psychotherapy and prosocial behavior, finds that people are more likely to learn from and/or sustain engagement in activities that they do for more self-determined reasons [51]. On the other hand, activities performed for less self-determined reasons (i.e., amotivated, external, and introjected motivations) are likely to be short-lived and have little impact on participants [52]. For instance, people who engage in pro-environmental behaviors like recycling or saving energy for highly self-determined reasons (e.g., because they enjoy doing so, or because doing so aligns with their internalized vision of themselves as a conservationist) engage in more pro-environmental behaviors, and are more likely to sustain this behavior, than people who engage in these behaviors for less self-determined reasons (e.g., because they would feel guilty if they didn’t recycle [53–55]). In the context of education, students taught using an externally motivating pedagogy that relies on threats, rewards, or shame lose interest, initiative and persistence in learning, and learn less effectively, compared to students taught in a manner that supports more self-determined motivations [56–58].

Importantly, SDT posits that motivations are malleable, and that a person’s motivations can become more self-determined if they find that an activity meets what SDT proposes are three fundamental human needs: competence, relatedness, and autonomy [50]. Competence refers to the need to feel effective and capable of meeting challenges. Relatedness refers to the need to belong and feel valued by others in a community. Autonomy refers to the need to feel that your behavior is self-directed and that you have freedom in the choices that you make. If an activity supports these three fundamental needs, a participant is more likely to experience self-determined motivation and, thus, to gain more from the experience.

A number of studies have examined motivation in the context of citizen science [59]. This body of work finds that most citizen scientists participate for highly self-determined reasons, including intrinsic motivations such as participating because it is exciting, interesting, or enjoyable, and identified motivations such as participating because one values getting to contribute to science or help the environment [60–65]. Less self-determined motivations are rarely measured and sometimes deliberately excluded from analyses of volunteers’ motivations; one study excluded external “pressure” as a motivation because the researchers doubted that this motivation was relevant to volunteer citizen scientists [45], and another study excluded motivational data from students who participated in a project because they were required to [36]. When less self-determined motivations *are* measured, they are typically, though not always, rated by volunteers as less important than more self-determined motivational factors. This includes externally regulated factors such as participating in order to compete on leaderboards [66,67], to receive something in return [68,69], to improve job prospects [70], to gain knowledge [71], and to avoid negative consequences [72]. It also includes introjected factors such as to gain recognition and reputation [73–75], or because one feels obligated to contribute [76–78]. Studies of associations between citizen scientists’ motivation and participation patterns find that volunteers with more self-determined motivations to contribute to citizen science tend to engage more deeply in projects [79] and provide more sustained – and more novel – contributions [45,80].

Importantly, few of these studies used the SDT motivational continuum explicitly when measuring motivation and so we have applied these labels *a posteriori* based on our interpretation of the factors and items used. Studies of citizen science motivation that do focus on SDT include various studies evaluating the three fundamental needs predicted by SDT: whether citizen science fulfills these needs [45], how failing to fulfill these needs leads to participant drop-out [81], and whether making design changes to meet these needs improves a project [82]. Perhaps the most comprehensive treatment of SDT in the context of citizen scientists is Rutten et al. [48], who reviewed the literature on motivation to map citizen scientists’ reported motivations onto the SDT continuum. Recognizing this gap in citizen science motivational studies, researchers have called for more studies investigating citizen science volunteer motivations using the framework of SDT [83].

### Objectives

While various researchers have suggested that partnerships between citizen science projects and facilitator organizations could facilitate deeper engagement by diverse volunteers [28], few studies have tested this explicitly and none have evaluated the importance of different types of motivation, particularly less self-determined motivations, for members of facilitator organizations. Studying members of facilitator organizations provides a unique opportunity to test enduring questions about citizen science volunteer retention, participation equity, and motivation.

For this research, we studied volunteers who participated in citizen science through a corporate social responsibility program. Specifically, in 2019, SciStarter.org, an online citizen science project clearinghouse, began a partnership with a large Fortune 500 company to create a customized online citizen science portal for their corporate volunteer program (to protect the anonymity of participants, we are unable to share the name of the company in this publication). Through this partnership, employees are able to find and contribute to citizen science projects that are hosted on the SciStarter portal. Participation in the program is voluntary, but highly encouraged by the company in several ways. For instance, certain employees are selected as leaders in promoting participation in the program and progress towards program goals (total number of volunteer hours, etc.) are tracked and publicized by the program. Within the context of this corporate volunteer program, our study had four objectives, with the overarching goal of better understanding the role of facilitator groups and motivations in connecting new audiences with the benefits of citizen science participation. We achieved each of these objectives in this study.

1. Characterize the overall participation of volunteers in the citizen science program.
2. Evaluate whether these corporate volunteers are more diverse than typical citizen scientists.
3. Evaluate the motivations of these corporate volunteers using the SDT motivational continuum and explore socio-demographic differences in motivations.
4. Identify factors, including motivations, associated with volunteers’ depth of engagement in the program.

## Methods

We used SciStarter digital metadata and a survey to study participation in the corporate citizen science program. All data used in this paper came from these sources. The human subjects research in this article was approved by the North Carolina State University Institutional Review Board under protocol no. 20934. We outline both data sources in more detail below.

### Digital metadata

SciStarter collects digital metadata about volunteer participation in the projects on its corporate volunteer portal using an Application Programming Interface (API). We downloaded this contribution data for each corporate volunteer from the start of the citizen science initiative in November 2019 through August 2022, when we began analyzing the data (i.e., nearly three years of contributions). From this data, we were able to derive detailed quantitative data about each corporate volunteer’s participation in the citizen science program. All digital metadata was shared with the authors in accordance with SciStarter’s privacy policy.

### Survey data

We surveyed a sample of the participants in the corporate citizen science volunteer program between April 1, 2020 and January 31, 2022 in order to collect socio-demographic data and data on volunteer motivations. The survey distribution protocol prioritized surveying volunteers before they began participating in the citizen science program. Links to the survey were posted on SciStarter’s online portal for corporate volunteers in April 2020 (approximately five months after the citizen science program launched) and remained there until April 30, 2021. Additionally, in May 2020, we sent emails to 490 employees who had already created a SciStarter account encouraging them to take the survey. We also sent automated emails promoting the survey to any employees who signed up for a SciStarter account between May 2020 and January 2022. A maximum of two reminder emails were sent to employees who did not complete the survey. Lastly, we shared a link to complete the survey during webinars introducing employees to citizen science. Employees who completed the survey were entered into a drawing for a $100 gift card in order to encourage participation. Written informed consent was required from participants before starting the survey.

In total, we received 459 responses to this survey. We eliminated substantially incomplete surveys (respondents who answered <10% of questions), survey responses that were clearly rushed (completed in less than 60 seconds), surveys with substantial straightlining, and three duplicate surveys (we eliminated the later response for each of these) for a final survey sample of 388. We were unable to calculate a precise response rate for this sample, as we did not know exactly how many volunteers saw the invitation to participate in the survey given the variety of channels through which the survey was promoted. However, given the total population of corporate citizen science volunteers (13,425; see below), this sample represents approximately 3% of the volunteer population, though 12% of employees who completed the survey did not go on to participate in the citizen science volunteer program (see below).

The survey had three primary goals. First, we evaluated participants’ prior citizen science experiences. To do so, we asked respondents whether they had experience participating in citizen science and, if so, to indicate what project(s) they had participated in and how frequently they participated. Second, we collected socio-demographic data from participants. Specifically, we asked respondents to indicate their gender, race/ethnicity, age, the level of education they had completed, whether they worked in science and technology-related fields (STEM), and their political ideology on a five-point scale from very liberal to very conservative. Third, we evaluated participants’ motivations for contributing to citizen science. We did so by adapting established instruments for assessing motivation along the SDT continuum to the context of citizen science. Respondents were asked why they were “participating in (or considering participating in) citizen science?” Fifteen items assessed volunteers’ motivations, with three items each for intrinsic regulation (e.g., “because I simply enjoy participating in the projects”), identified regulation (e.g., “because I really value getting to contribute to science”), introjected regulation (e.g., “because I’d feel bad if I didn’t do citizen science”), extrinsic regulation (e.g., “because it’s what I’m supposed to do”), and amotivated regulation (e.g., “I feel that contributing to citizen science is a waste of time”). The items used in the survey were adapted from the Learning Self-Regulation Questionnaire [84] and the Motivation Toward the Environment Scale [54]. Items were presented in a random order. Each item was assessed using an 11-point Likert scale from “Not at all agree” (0) to “Strongly agree” (10).

Respondents also answered a free-response question assessing their motivation for participating in citizen science (“Why are you participating in (or considering participating in) citizen science?”). This question preceded the Likert-type questions in order to avoid biasing responses. The survey also asked a variety of questions unrelated to this study (volunteers’ connection to nature, etc.). We used volunteers’ email addresses from the survey to link survey data to the digital metadata.

### Analysis

To assess volunteer participation in the program (Objective 1) we used the digital metadata to calculate the number of volunteers who participated in the corporate citizen science program, the number of volunteer hours spent on each project, and the number of projects volunteers participated in. We also calculated individual-level participation in the program in terms of each volunteer’s hours of participation, number of projects, and number of contributions (i.e., the number of times a volunteer participated in a given project on a given day). Next, we categorized each project according to its discipline (i.e., ecology/environment, health/medicine etc.) and mode (online or offline) using multirater-consensus categorizations employed in a previous publication analyzing projects hosted by SciStarter [5]. We then calculated the number of projects from each discipline and mode, and the number of volunteer hours spent on each discipline and mode. Lastly, in order to evaluate the representativeness of the survey sample, we used Wilcoxon signed-rank tests and Chi-square tests to compare the participation patterns of the full population of corporate volunteers to the participation of volunteers who took the survey [85].

We used the survey data to report descriptive statistics of the socio-demographics of the volunteers in the corporate citizen science program (Objective 2). We used Chi-square tests to compare these socio-demographics to the socio-demographics of SciStarter volunteers who aren’t members of a facilitator organization, which we assessed in a prior study [5]. We also compared these socio-demographic factors to the socio-demographics of the United States population (most of these corporate volunteers are based in the United States, as are most projects hosted by SciStarter).

We used the survey to conduct a quantitative assessment of the motivations of these corporate volunteers (Objective 3). We performed exploratory factor analysis (EFA) on the 15 motivation items in order to assess the validity of the proposed constructs using the “psych” package in R [86]. We opted for EFA rather than confirmatory factor analysis because these items were modified from their original instruments and had not been used in the context of citizen science before. We used the Kaiser Criterion (counting the number of eigenvalues greater than one) to establish the number of factors specified in the EFA [87] and used oblique rotation and Principal Axis Factoring. We calculated the aggregate mean score for each factor resulting from EFA by averaging the items that made up each factor for each respondent. We then calculated a “Relative Autonomy Index” (RAI) score for each respondent. The RAI is a single measure of self-determined motivation made by collapsing multiple motivation factors and is often used in studies utilizing SDT [88]. We calculated the RAI by summing the mean motivation scores for each volunteer and weighting each factor according to its degree of self-determined motivation (i.e., Autonomous – Controlled – Amotivated*2; see below). We then rescaled the RAI so that its scale was the same as the three individual motivation factors (i.e., had a range of zero to ten). Respondents who completed fewer than half the items in a given factor were not given an average score (~8% of respondents for each factor). We use quotes from the free-response motivation question to provide more context to the quantitative data. Next, we used multiple regression to explore whether the socio-demographic factors measured in the survey (race/ethnicity, gender, education, political ideology, STEM occupation and age) predicted volunteers’ self-determined motivation as assessed by the RAI motivation index using the “lm.beta” package in R [89].

To explore links between self-determined motivation and participation levels among corporate volunteers (Objective 4), we first calculated the association between volunteers’ mean score for each motivation factor and the number of hours they spent contributing to the citizen science program. We also calculated associations between volunteers’ RAI score and the number of hours they spent contributing to the program. Next, we used multiple regression to assess whether associations between motivation and participation were robust to other factors that might influence engagement in the citizen science program [89]. For this regression, the outcome variable was the total number of estimated hours a volunteer spent doing citizen science over the sampling period (November 2019 – August 2022). Because a small proportion of volunteers contributed far more hours than most volunteers, we log-corrected this duration data to reduce skew as has been done in other studies of citizen science participation with highly skewed participation data [74]. We included the motivational factors identified in the EFA from Objective 3 as predictor variables. We controlled for the socio-demographic factors collected in the survey (race/ethnicity, gender, education, political ideology, STEM occupation and age) since socio-demographic factors are known to influence participation in citizen science [5] and motivation [90]. We also controlled for the date a volunteer took the survey, given the wide date range over which volunteers completed the survey, and controlled for whether or not a volunteer had experience participating in citizen science prior to taking the survey.

## Results

### Goal 1: Participation patterns

Over the nearly three year sampling period, 13,425 volunteers contributed 31,143 hours to citizen science through this corporate volunteer program. This volunteer total represents approximately 11% of the total workforce of this company. These volunteers’ total hours of work equate to approximately $990,000, according to the US national volunteer hourly in-kind rate [4,91]. Individual volunteers’ hours of contributions ranged from 15 minutes to 381 hours. As with much citizen science [79], participation was highly skewed; the average volunteer participated for just 2.3 hours, the median volunteer participated for 1 hour, and the volunteers in the top 20th percentile of volunteer hours contributed 64% of the total hours. Approximately 59% of volunteers participated for an hour or less (*N *= 7,951) and just 6% of volunteers contributed more than five hours total (*N* = 826).

Volunteers who participated in the program made between 1 and 535 individual contributions to citizen science, with the average volunteer making 2.9 contributions and the median volunteer contributing once (“contribution” in this case means a volunteer participated in a given project on a given day; this definition is used because the amount of effort needed to submit a single data point varies widely from project to project). Contributions were inconsistent across the sampling period; for instance, contributions spiked in April of each year, presumably in conjunction with “Citizen Science Month” initiatives organized by SciStarter each April. Individual corporate employees who participated in the program contributed to between 1 and 18 different projects, with the median volunteer contributing to one project and the mean volunteer contributing to 1.4 projects. Approximately 62% of volunteers contributed just once, 11% of volunteers made multiple contributions to a single project and 27% of volunteers contributed to multiple projects (Table 1).

**Table 1:** Citizen science participation patterns of corporate employees according to population-level data for the entire volunteer sample (*N *= 13,425) and survey data from a smaller subset of volunteers (*n *= 343).

Participation pattern	Proportion of volunteer population	Proportion of volunteers who took survey^a^
Total duration of participation^**^ (Cramer’s V = 0.03)		
1 hour or less	0.59 (*N* = 7,951)	0.52 (*n *= 177)
>1–5 hours	0.35 (*N *= 4,648)	0.38 (*n *= 130)
>5–10 hours	0.04 (*N *= 503)	0.07 (*n *= 23)
>10 hours	0.02 (*N *= 323)	0.04 (*n *= 13)
Multi-project participation^**^ (Cramer’s V = 0.04)		
Single contribution to single project	0.62 (*N *= 8,296)	0.53 (*n *= 181)
Multiple contributions to single project	0.11 (*N *= 1,538)	0.10 (*n *= 33)
Multiple contributions to multiple projects	0.27 (*N *= 3,591)	0.38 (*n *= 129)

^a^Excludes volunteers who did not go on to participate in citizen science (12% of surveytakers).

**Indicates significant difference in participation patterns between volunteer population and survey sample (Wilcoxon signed-rank test (duration) and Chi-square test (multi-project participation); *p *< 0.01).

Volunteers participated in 49 different citizen science projects overall. Approximately half of these projects could be completed entirely on a computer and approximately half of the projects were related to ecology and the environment. There was a large amount of project-specific participation skew, with volunteers contributing fewer than 10 total hours to 17 of these projects and contributing more than 1,000 hours to seven different projects (Table 2). Health-related projects were the most popular (53% of all volunteer hours), followed by ecology and environment-related projects (22% of hours) and then geology and earth science-related projects (14% of hours). Approximately 65% of volunteer hours were spent on online-only projects, while 35% of volunteer hours were spent on projects with an offline component.

**Table 2:** The ten most popular projects for participants in the corporate citizen science volunteer program.

Project name	Total hours	Discipline	Mode
Neureka	5,221	Health and Medicine	Online
Stall Catchers	5,064	Health and Medicine	Online
GLOBE Clouds	3,527	Geology and Earth Science	Offline
Outbreaks Near Me	3,407	Health and Medicine	Online
Eureka Covid-19 Citizen Science	2,387	Health and Medicine	Online
iNaturalist	1,223	Ecology and Environment	Offline
GLOBE Trees	1,045	Ecology and Environment	Offline
EteRNA	892	Cell and Molecular	Online
ISeeChange	828	Ecology and Environment	Offline
Globe at Night	702	Pollution	Offline

Among the 388 employees who completed the survey, 85% had no experience with citizen science prior to taking the survey, and just 3% of volunteers had done citizen science more than once prior to taking the survey. Of these 388 surveyed employees, 88% would go on to participate in citizen science (per the digital metadata). Volunteers who took the survey participated in more citizen science, and were more likely to participate in multiple projects, than volunteers who did not take the survey (Table 1). However, the effect size of taking the survey on citizen science participation was small (Cramer’s V ≤ 0.04).

### Goal 2: Volunteer socio-demographic diversity

The demographic summaries reported below only include the 343 survey-takers who would go on to participate in the citizen science program (Goal 2 focuses on the diversity of citizen science *participants* rather than *prospective* participants; in addition, there were no statistically significant differences between volunteers who did and did not go on to participate in citizen science according to any of the socio-demographic variables we collected). Among these respondents, 60% identified as non-Hispanic White, 55% identified as women and the average age was 44 years old. Approximately 28% of survey-takers reported having a graduate, professional or other advanced degree, 53% of survey-takers had a bachelor’s or associate’s degree as their highest degree, and 19% of volunteers had at most a high school degree (this category includes individuals with some postsecondary education but no postsecondary degree). A plurality of respondents had moderate political views (46%) and a majority of respondents reported working in STEM-related fields (62%; Table 3). Corporate volunteers were more representative of the US average in terms of their race/ethnicity, gender and level of education than typical SciStarter volunteers (i.e., those not involved in the corporate volunteer program), who tend to be White, highly educated women (Table 3). In addition, while there is no existing data on the political beliefs of typical SciStarter volunteers specifically, these corporate volunteers’ political beliefs were more representative of the US average than those of citizen scientists from other domains, who tend to identify predominantly as Liberal [5,92]. Corporate volunteers were less representative of the US average than typical SciStarter volunteers in terms of their age and whether they worked in science-related fields, with these corporate volunteers less likely to be above retirement age and more likely to work in STEM.

**Table 3:** Summary socio-demographic data for corporate volunteers, typical citizen science volunteers on SciStarter [5], and the general US population.

Demographic	Corporate volunteers (*n *= 343^a^)	Typical SciStarter volunteers (*n *= 423)	US population^b^
Race/ethnicity^***^			
White	0.60	0.88	0.58
Black	0.12	0.03	0.12
Asian	0.12	0.02	0.06
Hispanic	0.10	0.04	0.19
Other	0.06	0.03	0.05
Gender^***^			
Women	0.55	0.69	0.50
Men	0.45	0.31	0.50
Age^***^			
≥ 65	0.02	0.18	0.17
< 65	0.98	0.82	0.83
Highest degree^***^			
≤ High school	0.19	0.09	0.56
Associate’s/bachelor’s	0.53	0.37	0.30
Graduate/professional	0.28	0.53	0.14
Politics^c^			
Liberal	0.35		0.24
Moderate	0.46		0.38
Conservative	0.19		0.37
Occupation^***^			
STEM	0.62	0.48	0.07
Not STEM	0.38	0.52	0.93

^a^Excludes survey-takers who did not volunteer. Non-responses (19–31% for each variable) not included in proportions.

^b^Data from US Census Bureau [93–95], Gallup [96], and US Bureau of Labor Statistics [97].

^c^Survey of typical SciStarter volunteers did not collect data on political views.

***Chi-square test comparing corporate volunteers and typical volunteers; *p* < .001

### Goal 3: Volunteer motivations

EFA indicated that the motivation items mapped to three factors rather than five, according to the Kaiser Criterion. One item from the proposed external regulation factor had a loading of only 0.3, below the threshold of 0.4 for interpretability established by Stevens [98], and didn’t load on any of the other factors, so we removed it from the analysis. All other items had factor loadings of at least 0.4. We repeated the EFA with the problematic item removed and the items again mapped onto three factors (Table 4). The first factor, “Autonomous Motivation” (Cronbach’s α = 0.91), included all three items relating to intrinsic regulation, all three items relating to identified regulation, and one item from introjected regulation. The second factor, “Controlled Motivation,” contained two items from the proposed introjected regulation factor and two items from external regulation (α = 0.70). The third factor, “Amotivation” (α = 0.67) contained all three remaining items relating to amotivated regulation.

**Table 4:** Means and loadings across three factors for volunteers’ motivation to participate in citizen science (n = 388).

Item	Mean	SD	Factor loadings
Autonomous Motivation (*α* = 0.91)	7.82	1.87	
Because I think it is a good idea to participate in citizen science	8.27	2.22	0.9
Because doing citizen science is a useful way to help society	8.04	2.26	0.8
For the pleasure I get in learning new things about science and nature	8.13	2.08	0.8
Because I really value getting to contribute to science	7.92	2.25	0.8
Because participating in the projects is fun and interesting	7.85	2.22	0.7
Because I’d feel proud of myself if I contribute to citizen science	7.55	2.62	0.7
Because I simply enjoy participating in the projects	6.96	2.61	0.6
Controlled Motivation (*α* = 0.70)	4.31	2.53	
Because I’d feel bad if I didn’t do citizen science	3.34	3.27	0.8
Because I think I’d regret not trying citizen science	4.99	3.51	0.7
Because it’s what I’m supposed to do	4.74	3.54	0.5
Because my friends or coworkers encouraged me to	4.20	3.70	0.4
Amotivation (*α* = 0.67)	1.83	2.18	
I can’t see what’s in it for me	1.86	2.82	0.7
I can’t see how my efforts are helping science	2.43	3.03	0.6
I feel that contributing to citizen science is a waste of time	1.22	2.58	0.5

Each question used an 11-point Likert scale from 0 (“Not at all agree”) to 10 (“Strongly agree”).

Overall, responses to the quantitative motivation items indicated that corporate volunteers have relatively high levels of self-determined motivation to participate in citizen science. On the 0–10 scale used in the survey, scores for the Autonomous factor (high self-determination) were high (x̄ = 7.82), scores for the Controlled factor (low self-determination) were moderate (x̄ = 4.31) and scores for Amotivation were low (x̄ = 1.83; Table 4). The mean score on the RAI was 6.61 (range = 0–9.86).

Responses to the qualitative motivation question indicated a range of motivations. Some volunteers described highly intrinsic motivations, such as: “*it seems interesting*”, “*Honestly, the study sounds pretty neat!*” and “*Enjoy doing it*”. Likewise, many responses indicated that volunteers participated because doing so aligned with their internals values (i.e., identified regulation) such as: “*Interested in helping scientists solve existential world problems*” and “*Looking for ways to volunteer and give back to my community*”. Many of these volunteers identified specifically with the health-related or environment-related focus of many of the projects, for instance: “*I joined the first study as part of a volunteer program through my employer and since I am a high risk individual, I felt i could contribute quite a bit to the study of the virus*”, “*Was interested due to the dementia part. I have family that suffer and suffered from it.*”, and “*Pollinators are important to our ecosystem. I want to help provide a read of the pollinators in my area*.” A few volunteers said they participated for introjected reasons, such as one respondent who said, “*My daughter is fascinated by science and I’m trying to model good behaviors of involvement for her*.” Finally, some responses indicated highly external motivations for participating, such as: “*It’s part of my volunteer day with work*.”, “*assigned to do so through my employer*”, “*for work requirement*”, and “*Getting credit at work for volunteering*”. A number of volunteers indicated that multiple different motivations along the SDT motivational continuum were operating simultaneously, such as a respondent who said “*Need to commit a specific set of hours today, and the project sounded interesting*.”

The only socio-demographic variable that was significantly predictive of volunteers’ self-determined motivation was gender, with women scoring, on average, approximately 0.9 points higher on the 0–10 RAI scale (S1 Table).

### Goal 4: Motivations and other determinants of depth of engagement in citizen science

**Fig 2:**
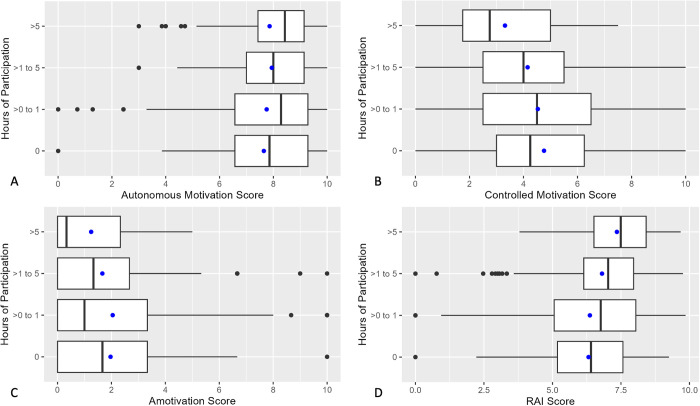
Associations between volunteers’ motivations for participating in the citizen science program and the number of hours they spent contributing to projects. Figure is separated into panels for autonomous (A), controlled (B), and amotivated (C) motivations. Panel (D) depicts the association between hours of participation and a “Relative Autonomy Index,” which combines the three motivation factors into a single measure of self-determined motivation. Vertical black line denotes median, blue dot denotes mean (**n* *= 356).

Self-determined motivation was associated with more hours of participation in citizen science (Fig 2). Specifically, higher scores on the Autonomous Motivation factor (2a) and the RAI (2d) were associated with more participation, while higher scores on the Controlled motivation (2b) and Amotivation factors (2c) were associated with fewer hours of participation. For these analyses, we binned total hours of participation to increase readability owing to the right-skew of the variable.

**Fig 3:**
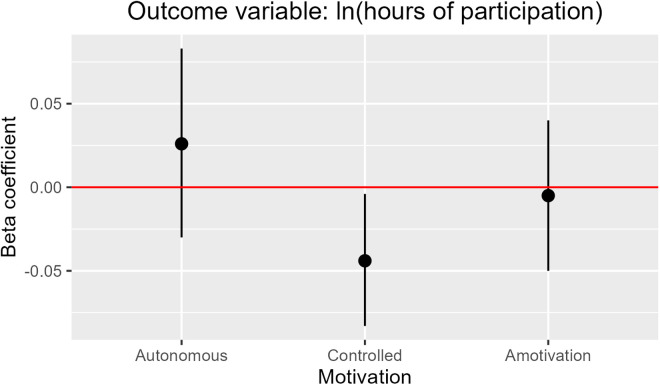
Model of the effects of volunteer motivation on the number of hours spent contributing to citizen science (log-corrected). Regression model controls for volunteers’ race/ethnicity, gender, education, political beliefs, age, whether they worked in STEM, when they completed the survey and whether they had prior citizen science experience. Figure shows 95% confidence intervals for the three motivation variables (vertical black lines). Degrees of freedom = 221; adjusted r^2^ = 0.11.

When controlling for socio-demographic variables, survey completion date, and volunteers’ prior citizen science experience, higher scores on the Controlled motivation factor had a significant association with fewer hours of participation in citizen science (Fig 3). Specifically, a one-point increase in mean controlled motivation, as measured on the 0–10 scale, predicted approximately 4% fewer hours of participation in the citizen science program. Thus, a volunteer who “completely agreed” that they participated in citizen science “because it’s what I’m supposed to do” (i.e., scored a “10” on this motivation item) and had similar scores for the other Controlled motivation items would be expected to spend 1.2 fewer hours contributing to citizen science than a volunteer who did not agree at all with these motivations (i.e., scored a “0” on these items) and participated for three hours. Higher scores on the Autonomous factor were associated with more participation in citizen science, but the relationship was not statistically significant. The Amotivation factor did not appear to have a relationship with the number of hours a volunteer spent contributing to citizen science.

Two of the socio-demographic variables in the model also had a significant relationship with the number of hours a volunteer spent participating in citizen science. Specifically, Black volunteers participated in, on average, 25% fewer hours of citizen science than White volunteers, and a one-year increase in volunteer age was associated with an average 1% increase in the time volunteers spent participating, controlling for the other factors in the model. In addition, the 15% of volunteers with citizen science experience prior to taking the survey participated in 57% more citizen science than volunteers without this experience. Standardized beta coefficients (*β*) show that having prior citizen science experience was the strongest predictor of total hours of participation in the citizen science program, followed by age, controlled motivation, and identifying as Black (Table 5).

**Table 5:** Results of regression predicting hours of citizen science contributions (log-corrected) from volunteers’ motivations, socio-demographics and other factors.

Variable	*B*	Standard error	*β*
Motivations			
Autonomous	0.026	0.028	0.067
Controlled	−0.044^**^	0.020	−0.158^**^
Amotivation	−0.005	0.023	−0.016
Socio-demographics			
Race/ethnicity [reference: White]			
Asian	0.077	0.148	0.035
Black	−0.289^**^	0.138	−0.140^**^
Hispanic	0.071	0.150	0.031
Other	−0.235	0.179	−0.085
Man	−0.149	0.092	−0.107
≥ Bachelor’s Degree	−0.004	0.096	−0.002
Liberal	0.095	0.093	0.066
STEM Occupation	0.028	0.096	0.019
Age	0.012^**^	0.005	0.163^**^
Other factors			
Survey completion date	0.000	0.000	0.055
Has citizen science experience	0.454^***^	0.132	0.220^**^
Constant	−5.334	6.833	NA

We report both unstandardized (*B*) and standardized (*β*) coefficients for each predictor (221 degrees of freedom; adjusted r^2^ = 0.11; ^**^p < 0.05; ^***^ *p *< 0.01).

## Discussion

This study found that a corporate volunteer program has enabled more than 13,000 people to contribute tens of thousands of hours to dozens of citizen science projects across a variety of disciplines and modes, representing an-kind service donation of nearly $1 million USD. The vast majority of these volunteers were new to citizen science. While most of these volunteers dabbled in citizen science and contributed just once or twice, hundreds of volunteers became dedicated citizen scientists, making many contributions and often participating in more than one project.

We found that this corporate volunteer cohort was much more socio-demographically representative of the US population than typical citizen scientists, with participation by racial/ethnic minorities (40% of all volunteers) approximating these groups’ overall representation in the US population, and with approximately equal participation by men and women. In addition, while the average corporate volunteer was more educated and more politically Liberal than the general US population, this skew was not nearly so pronounced as among typical citizen scientists. While the proportion of corporate volunteers over retirement age, as well as the proportion of volunteers with STEM careers, was less representative of the US population than typical citizen scientists, this was expected given that volunteers were employed (i.e., not retired) and worked for a technology-related company. The racial/ethnic diversity of the corporate volunteers was particularly striking given how overwhelmingly White most citizen science programs are [5]. Taken together, these results indicate that facilitator organizations like corporate social responsibility programs appear to be a promising source of volunteers for citizen science projects looking to connect with volunteers who are new to citizen science, who come from more diverse socio-demographic backgrounds, and who therefore might be less self-selecting than typical volunteers. Thus, these corporate volunteers might have more to gain from participation, such as through knowledge gains and changes in environment-related or science-related attitudes and behaviors.

Although these programs represent a unique opportunity to increase diversity and inclusion in citizen science, organizations hoping to increase the diversity of their participants need to look beyond simply requesting participation from new volunteers—the “pipeline” or “pathway” approach to diversifying engagement in science [26]. Instead, projects must first evaluate the culture of projects, examine how that culture is experienced by their volunteers, and take steps to make that culture more inclusive [2]. The fact that most volunteers soon dropped out of the program after participating once or twice suggests that there are persistent barriers preventing deeper engagement in projects, and associated learning outcomes, among these volunteers.

One such barrier is motivation. While many corporate volunteers were motivated to participate in citizen science for self-determined reasons (e.g., because it’s enjoyable or because the volunteer deems that it is important), others were motivated by less self-determined, controlling factors such as social pressure and guilt. Thus, even though most volunteers identified with intrinsic rationales for participating, the structured nature of the corporate volunteer program, through which participation is encouraged, or even coerced, led some volunteers to be motivated by external factors—something both the quantitative and qualitative survey responses made clear. According to our analysis, men may be particularly prone to participating as a result of these less self-determined motivations. Although other studies have examined citizen science motivation through the lens of SDT [45,48], few have specifically explored the role of extrinsic motivations or the potential for dynamic shifts in motivation over time.

Being driven to participate in citizen science by controlled factors (e.g., participating “because it’s what I’m supposed to do”) predicted significantly less engagement in the citizen science program. While this effect size was relatively small, small differences can add up to big effects across thousands of volunteers. More autonomous motivation (e.g., participating because it’s “fun and interesting”) was associated with higher levels of participation in the program, though this relationship was not statistically significant in our regression model. Amotivated rationales for participating in the citizen science program didn’t appear to have a clear relationship with participation duration, though the direction of the effect was in the direction we anticipated (more amotivation was associated with less participation). Clearly, not all motivations are the same; someone acting on external factors as a rationale for participating in citizen science is more likely to drop out of the program than someone driven by more autonomous motives. Taken together, these findings support a central tenant of Self-Determination Theory—that less self-determined motivations for doing an activity lead to lower-impact experiences [51]—and demonstrate the enduring importance of self-determined motivation in sustaining behavior.

The impact of self-determined motivation on participation provides an important lesson for citizen science administrators: building an impactful, self-sustaining citizen science program requires volunteers who are inherently interested in and committed to a project rather than simply contributing out of a sense of obligation. As facilitator organizations take on a larger role in connecting volunteers to citizen science projects [31], this lesson will become increasingly important. With more volunteers contributing, in part, due to group pressure or requirements, leaders of citizen science projects must design projects so that they tap into these volunteers’ self-determined motivations (see “Implications for Practice” below). While many corporate volunteers had at least moderately high levels of self-determined motivation, it’s possible that other facilitator organizations attract volunteers with very low levels of self-determination. For instance, some students whose participation is compelled by their schools apparently have such little intrinsic motivation to participate that they falsify data to avoid contributing time and effort to a project [99]. Project owners and facilitators should be attuned to the motivations of their particular volunteers to inform the design of impactful programs [59].

Importantly, motivation was not the only factor correlated with participation depth in citizen science among the volunteers in our study. The most important determinant of participation duration was prior experience with citizen science, suggesting that volunteers’ initial experiences with citizen science could provide some degree of familiarity with the participation process that made further participation more likely [81]. However, it’s difficult to interpret this factor effectively because some volunteers’ prior participation in citizen science was in the corporate program, and this participation was itself captured in the outcome (hours of participation in citizen science).

Volunteers’ racial identity was also a significant determinant of participation, with Black volunteers participating in 25% fewer hours than White volunteers, controlling for the other variables in the model. This suggests that even though Black volunteers have similar levels of self-determined motivation to participate in the projects as White volunteers (S1 Table), there are other factors—such as broader social inequities—limiting their participation specific to their socio-demographic identity. For example, as a result of historical and contemporary racism, Black individuals in the US have less privilege than White individuals [100] and individuals with more privileged positions in society have been shown to volunteer more, possibly because they have more resources and their immediate needs are met, and thus they can afford to spend more time engaged in an unpaid activity without obvious, tangible benefits [101,102]. In addition, historically, Black individuals in the US have been excluded from community volunteer organizations like the Elks and the Masons [103], an experience which may impact norms related to engagement in volunteering today. The lower engagement by Black volunteers in the citizen science program may also reflect the culture embodied by the types of projects offered by the corporate volunteer program. Bevan et al. [26] point out that STEM initiatives often fail to sustain engagement by diverse audiences because “the dominant cultural norms for engaging in STEM typically are the norms of the populations that have participated in and institutionalized STEM as we know it today” (p. 9). For instance, engagement in STEM is often characterized by a “mind-body duality” that separates reason and emotion, and this epistemology may be less familiar to people from communities that emphasize collective decision-making. If an individual doesn’t see their cultural norms or identity reflected in a practice, then they may choose to reject that practice [26]. This is a particularly salient point given the overwhelming whiteness of most citizen science programs [5,23].

Age was also shown to be a significant determinant of participation in the corporate citizen science program, with older volunteers likelier to contribute more hours. A number of studies have found that citizen science projects engage a disproportionately older segment of the population, and some have suggested that this is because retired people have more time to volunteer [23,24]. The present study shows that this trend holds even among volunteers that are all employed. One possible explanation for this finding is that older workers have more time to spend volunteering since they are less likely to have dependent children, which occupy the free time of younger workers. This pattern of deeper involvement in volunteering by older individuals often holds across other volunteering sectors [104].

Men in our study had lower levels of self-determined motivation to participate in citizen science than women. This finding reflects other studies of volunteer motivations across broader sectors, which suggest that women often report stronger intrinsic motivations to volunteer than men [105,106]. However, men in our study did not participate in fewer hours of citizen science when controlling for motivation. Studies have shown that gender differences in citizen science participation rates are inconsistent, with some projects attracting more women and others tending to attract more men [23]. Collectively, these results suggest that studies assessing gender-based differences in volunteering on citizen science engagement should account for motivation when trying to isolate a gender-based effect.

Importantly, the findings from this study may not be representative of volunteers from other facilitator organizations [28]. Future work should investigate the diversity and motivations of volunteers from facilitator organizations from schools, churches, and other corporate volunteer programs. In addition, more comparative, empirical studies are needed that evaluate how projects are meeting volunteers’ needs and the links between meeting needs, motivation, and volunteer retention. More longitudinal studies are also needed that track shifts in volunteer motivations and outcomes over time [59]. Finally, future studies, particularly qualitative studies, should identify what specific barriers limit engagement in citizen science by specific groups, such as Black individuals or younger volunteers.

### Implications for practice

Facilitator organizations such as corporate volunteer programs offer a promising source of new citizen science volunteers who can provide important contributions to science, who have more to gain from their experiences (e.g., new experiences with science) than conventional self-selecting citizen scientists, and who may improve the practice of citizen science by bringing new, more diverse perspectives and approaches. However, achieving these goals requires sustained engagement in citizen science, something that our study found was relatively rare among a cohort of corporate volunteers. Project owners and facilitator organizations should engage in reflective practice to assess whether their projects are supporting self-determined motivation [107].

In cases where volunteer groups have low self-determination, steps should be taken to change this. According to Self-Determination Theory, people develop self-determined motivation when their needs are met for competence, relatedness and autonomy [50]. We suggest a number of tangible ways that projects might meet these needs in Table 6. Meeting volunteers’ need for competence, for instance, might be met through comprehensive trainings and opportunities to track progress and demonstrate efficacy [82,108]. Competence might be a particularly salient factor for facilitator group volunteers, who, as we demonstrated in this study, may have less experience with citizen science and may lack confidence in their abilities to participate in science-related activities. Gamification can be another way to meet volunteer needs for competence, such as using points, leaderboards and digital badges, however this can have unintended negative consequences. If volunteers’ primary motivation revolves around the project’s gamified elements, then their intrinsic motivation can actually be undermined by these extrinsic factors through the process of “crowding out” [109]. Used effectively, gamified and/or competitive elements of participation reinforce interest in and commitment to project goals rather than distract from them.

**Table 6:** Suggestions for designing citizen science projects to facilitate self-determined motivation by meeting volunteers’ needs for competence, relatedness and autonomy, using examples from popular projects among corporate volunteers.

Need	Ways projects can meet need	Examples of project designs that meet this need
Competence: feeling effective and capable	Training modules; positive/ constructive feedback on participation (especially early participation); Ability to track progress and set/achieve participation goals; Ability to demonstrate efficacy (e.g., train less experienced volunteers, visualize increases in contribution amount/quality)	Zooniverse interactive tutorials; StallCatchers leaderboards; Ability to classify other users’ observations on iNaturalist; Progress bars/badges on different tasks on Neureka; Educational feedback to quiz responses on Neureka
Relatedness: feeling that one belongs in a community	Opportunities to interact with other participants, scientists, and projects owners, whether digitally and/or in person; Opportunities to build camaraderie/ a shared sense of purpose; Opportunities to see one’s identities reflected in project volunteers/facilitators	Ability to form teams on StallCatchers; Community-building reportbacks from Eureka Covid-19 project (i.e., a podcast); Chat feature on EteRNA; Ability to interact with others in one’s local area on iSeeChange; Forums on Zooniverse; ability to observe contributions of friends on iNaturalist; Crowd the Tap’s partnership with diverse facilitator organizations
Autonomy: feeling that one’s behavior is self-directed	Access to data to use for own creative purposes; Ability to follow curiosities and pursue answers to one’s own research questions; Flexibility in protocols such that volunteers can participate in a unique way that aligns with their individual interests	Ability to “favorite” observations on Zooniverse and save to a personal folder; ability to create one’s own projects within iNaturalist; Unstructured protocols on iNaturalist (can submit observations whenever/wherever/ of whatever species); Ability to search/visualize/ download data on Globe at Night; Ability to solve previously unsolved problems creatively on EteRNA (e.g., “Psuedoknot Challenge); Emphasis on individual narrative communication on iSeeChange

Volunteer needs for relatedness can be met by fostering volunteer community within projects such as through forums and other opportunities to interact with other volunteers and project scientists [110]. While many projects use reportbacks to communicate project results to participants (one-way communication), the need for relatedness is best met by opportunities to build relationships, such as through chat features, forums, in-person meetings and other forms of two-way communication. Relatedness needs might also be met by making projects more inclusive to people from different backgrounds, as discussed above, such that more volunteers see their identities reflected in other project volunteers and facilitators.

Meeting volunteer needs for autonomy can be met by providing volunteers access to project data, opportunities to follow their own scientific curiosities, and allowing for flexibility in how volunteers complete tasks and follow protocols [49,111]. Projects that rely on rigid protocols and that limit access to data are unlikely to meet volunteer needs for autonomy. Among those needs identified by Self-Determination Theory, the need for autonomy may be the least fulfilled by modern citizen science that often embraces a crowdsourced approach to data collection. While many historical citizen science projects involved dedicated amateurs pursuing their own curiosities through projects they created themselves (e.g., birdwatching enthusiasts initiating monitoring projects) most of today’s projects (and all the projects these corporate volunteers participated in) involve top-down protocols designed by and for professional scientists and scientific institutions [112]. These “contributory” style projects [113] inherently stifle some amount of volunteer autonomy, since volunteers are answering someone else’s research question rather than following their own curiosities or pursuing issues that are relevant to their lives. iNaturalist, which allows volunteers to create sub-projects based on local or taxonomic-based interests, as well as EteRNA, which poses previously unsolved challenges that volunteers solve creatively, provide some examples of how a few popular projects are fulfilling volunteers’ autonomy-related needs. Citizen science would better support volunteer autonomy if it served more effectively as a space where people can develop and test their own scientific questions, rather than solely contributing data to serve the research interests of professional scientific institutions. These suggestions should serve as a starting point for thinking more deeply about meeting volunteer needs to support self-determined participation in citizen science.

## Conclusion

Every year, citizen scientists provide a tremendous, voluntary service to science—an experience from which volunteers derive value. Stakeholders from across the citizen science landscape, including those involved with corporate volunteer programs, should recognize this effort by striving to support volunteer needs and intrinsic motivations and providing avenues for individuals from diverse communities to contribute to and gain from this growing global phenomenon. Meeting these needs for new populations of volunteers may entail re-envisioning some dimensions of modern citizen science practice itself and finding ways to cultivate personal agency and empowerment that lead to intrinsically-motivated actions.

## Supporting information

S1 Table Results of regression predicting self-determined motivation, as measured by the RAI, from socio-demographic factors of surveyed corporate volunteers. (DOCX)
