# Supplementary material for: The role of intrinsic motivation in sustaining citizen science participation among diverse participants in a corporate volunteer program
Source: PLoS One. 2025 Sep 5;20(9):e0331221. doi: 10.1371/journal.pone.0331221 (PMC12412928; doi:10.1371/journal.pone.0331221)
Supplement: S1 Table — (DOCX) [file pone.0331221.s001.docx]

**S1 Table. Results of regression predicting self-determined motivation, as measured by the RAI, from socio-demographic factors of surveyed corporate volunteers.**

| Variable | *B* | Standard Error | *β* |
| --- | --- | --- | --- |
| Race/ethnicity [reference: White] |  |  |  |
| Asian | -0.364 | 0.403 | -0.061 |
| Black | -0.602 | 0.375 | -0.108 |
| Hispanic | -0.049 | 0.412 | -0.008 |
| Other | -0.205 | 0.494 | -0.027 |
| Man | -0.853^***^ | 0.246 | -0.227^***^ |
| ≥ Bachelor’s Degree | 0.171 | 0.264 | 0.043 |
| Liberal | 0.464 | 0.252 | 0.119 |
| STEM Occupation | -0.126 | 0.262 | -0.032 |
| Age | 0.011 | 0.013 | 0.056 |
| Constant | 6.634 | 0.663 | NA |

We report both unstandardized (*B*) and standardized (*β*) coefficients for each predictor (226 degrees of freedom; adjusted r^2^ = 0.05; ^***^ *p* < 0.01).
